# The temporo-parietal junction contributes to global gestalt perception—evidence from studies in chess experts

**DOI:** 10.3389/fnhum.2013.00513

**Published:** 2013-08-28

**Authors:** Johannes Rennig, Merim Bilalić, Elisabeth Huberle, Hans-Otto Karnath, Marc Himmelbach

**Affiliations:** ^1^Division of Neuropsychology, Center of Neurology, Hertie-Institute for Clinical Brain Research, University of TübingenTübingen, Germany; ^2^Department of Neuroradiology, University of TübingenTübingen, Germany; ^3^Neurology and Neurorehabilitation Center, Luzerner KantonsspitalLuzern, Switzerland; ^4^Department of Psychology, University of South CarolinaColumbia, SC, USA

**Keywords:** gestalt perception, visual grouping, temporo-parietal junction, object perception, expertise, fMRI, simultanagnosia, chess

## Abstract

In a recent neuroimaging study the comparison of intact vs. disturbed perception of global gestalt indicated a significant role of the temporo-parietal junction (TPJ) in the intact perception of global gestalt (Huberle and Karnath, 2012). This location corresponded well with the areas known to be damaged or impaired in patients with simultanagnosia after stroke or due to neurodegenerative diseases. It was concluded that the TPJ plays an important role in the integration of individual items to a holistic percept. Thus, increased BOLD signals should be found in this region whenever a task calls for the integration of multiple visual items. Behavioral experiments in chess experts suggested that their superior skills in comparison to chess novices are partly based on fast holistic processing of chess positions with multiple pieces. We thus analyzed BOLD data from four fMRI studies that compared chess experts with chess novices during the presentation of complex chess-related visual stimuli (Bilalić et al., 2010, 2011a,b, 2012). Three regions of interests were defined by significant TPJ clusters in the abovementioned study of global gestalt perception (Huberle and Karnath, 2012) and BOLD signal amplitudes in these regions were compared between chess experts and novices. These cross-paradigm ROI analyses revealed higher signals at the TPJ in chess experts in comparison to novices during presentations of complex chess positions. This difference was consistent across the different tasks in five independent experiments. Our results confirm the assumption that the TPJ region identified in previous work on global gestalt perception plays an important role in the processing of complex visual stimulus configurations.

## Introduction

A crucial aspect of visual object recognition is the grouping of single elements to a global entity or so-called gestalt (Wertheimer, 1923; Koffka, 1935). The neuronal correlates of global processing or visual integration are still a matter of lively debates. Patients suffering from simultanagnosia, the inability to perceive a global gestalt first described as part of the Bálint syndrome (Bálint, 1909), typically show bilateral lesions in posterior parieto-temporal brain areas, whereas a remarkable variability concerning the exact localization is still prevalent (Rizzo and Hurtig, 1987; Friedman-Hill et al., 1995; Rafal, 1997; Karnath et al., 2000; Tang-Wai et al., 2004; Valenza et al., 2004; Huberle and Karnath, 2006, 2010; Thomas et al., 2012). Moreover, there is an inconsistency between functional imaging studies that attributed global perception to unilateral regions along the ventral visual pathway (Fink et al., 1996, 1997a,b) and other studies that found an association with posterior parietal and/or parieto-temporal areas (Yamaguchi et al., 2000; Himmelbach et al., 2009; Huberle and Karnath, 2012; Zaretskaya et al., 2013).

Research in chess experts provided a large body of data addressing neuronal correlates of visual skills (Bilalić et al., 2010, 2011a,b, 2012; Krawczyk et al., 2011). For research on object recognition and visual integration chess appears to be particularly suitable as it features various, clearly distinguishable individual objects that allow the composition of complex stimulus configurations with graded complexity. Furthermore, chess provides the opportunity to compare highly trained experts with novices based on a standardized rating system (Elo, 1978). Behavioral studies demonstrate that domain-specific knowledge, acquired through prolonged and focused training (Ericsson et al., 1993), enables experts to quickly grasp the essence of complex chess positions (DeGroot, 1978; Bilalić et al., 2008a). Instead of perceiving individual chess objects serially like novices, experts perceive meaningful units of several objects, called chunks (Chase and Simon, 1973) or templates (Gobet and Simon, 1996), which are linked with typical actions through pattern recognition mechanisms (Bilalić et al., 2008a,b, 2009, 2010). A typical chess position featuring numerous individual objects represents a single meaningful unit to chess experts. In a recent series of fMRI studies, Bilalić et al. (2010, 2011a,b, 2012) demonstrated that chess experts also showed different neuronal response patterns in the ventral visual system compared to novices. Typically, chess experts showed higher signal increases mostly in the temporal lobe compared to novices during the observation of chess stimuli. A study by Krawczyk et al. (2011) using comparable stimulus material revealed a similar result pattern with higher signals in temporal and frontal brain areas for experts compared to novices.

Based on the assumption that the behavioral advantage of chess experts is, at least partially, based on superior skills in the visual integration of multiple chess pieces we hypothesized that there should be a difference in the BOLD signal in regions that were functionally mapped in an independent study of global perception using substantially different stimulus material (Huberle and Karnath, 2012). In detail, the temporo-parietal junction (TPJ) was investigated by using an independent set of data from chess experts as well as novices. Several studies investigating neuronal processes of visual grouping used stimuli that may have evoked neuronal responses depending on low-level visual features like spatial frequencies of luminance changes (e.g. Fink et al., 1996; Huberle and Karnath, 2012). The stimuli examined in the ROI analyses of the present approach were substantially different from simple hierarchical Navon-like (Navon, 1977) stimulus material. The relationships between chess pieces that support the emergence of a global percept are not based on low-level visual features but on the knowledge about these pieces and their semantic relations. We compared signal levels in chess experts and novices in region of interest (ROI) analyses using four independent fMRI datasets taken from previously published studies on chess expertise (Bilalić et al., 2010, 2011a,b, 2012). We analyzed three ROIs defined by the data from Huberle and Karnath (2012). All three regions were located in the area of the right or left TPJ. While in three of these studies (Bilalić et al., 2010, 2011b, 2012) visual processing required an analysis of highly complex chess positions, one task (Bilalić et al., 2011a) focused on simple object perception.

## Materials and methods

### Participants

Eleven subjects (3 males/8 females; mean age 24.6 years, SD ± 0.7 years) participated in the study of Huberle and Karnath (2012). Subjects had normal or corrected to normal vision and reported no history of neurological impairment affecting their visual capacity. In all four studies of Bilalić et al. (2010, 2011a,b, 2012) expert as well as novice chess players participated (Table 1). Tournament players get rated based on their performance against other rated players. The international chess Elo scale is an interval scale with a theoretical mean of 1500 and standard deviation of 200 (Elo, 1978). Experts are players with a rating of 2000 Elo points or more. The experts included in the present studies were rated with an average around 2100 points. Novice players were hobby players who played chess occasionally. Their chess skills were clearly inferior to experts but they had no difficulties in identifying chess pieces and their functions. All players were male and right-handed. The Institutional Review Board of the Ethic Committee of Tübingen University approved both studies and written informed consent was obtained from all participants. All studies were performed in accordance with the ethical standards laid down in the 1964 Declaration of Helsinki.

**Table 1:** **Participants in the studies of Bilalić et al. (2010, 2011a,b, 2012): group, mean age, mean skill level as measured by the Elo rating (see Methods) with standard deviation (SD), number of standard deviations above the mean, and number of participants in each group in all four experiments**.

**Experiment**	**Group**	**Age ± SD**	**Elo ± SD**	**SDs above mean**	***n***
1	Expert	30 ± 2	2117 ± 53	3	7
	Novice	28 ± 1	−	−	8
2	Expert	29 ± 7	2130 ± 147	3	8
	Novice	29 ± 5	−	−	8
3	Expert	30 ± 2	2117 ± 53	3	7
	Novice	29 ± 1	−	−	8
4	Expert	30 ± 5	2108 ± 148	3	8
	Novice	29 ± 4	−	−	15

### Procedure and stimuli

**Figure 1:**
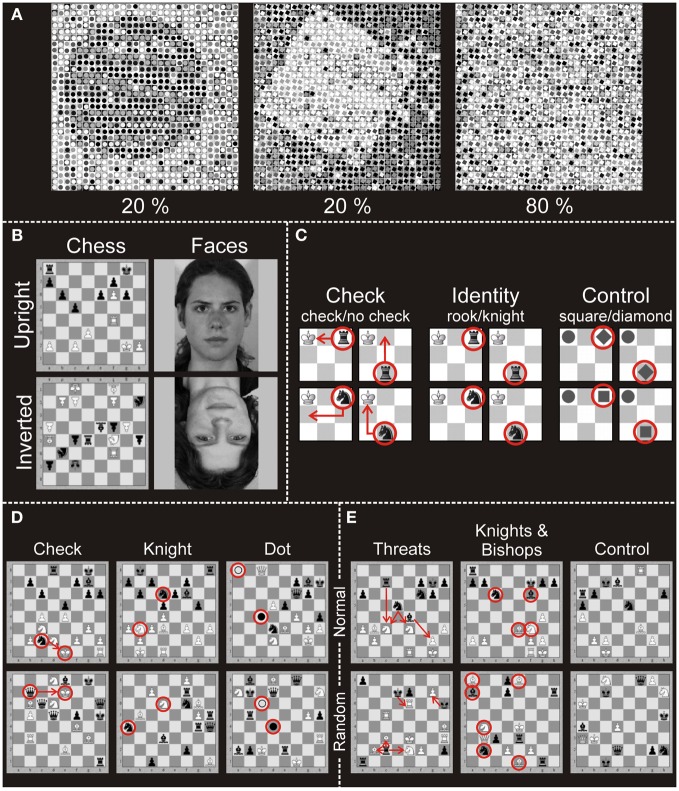
**Stimuli applied in the different experiments. (A)** Illustration of the global stimuli used in the experiment of Huberle and Karnath (2012). The pictures show two 20% scrambled global objects (circle, square; intact global perception) and one 80% scrambled global object (disturbed global perception). **(B)** Stimuli used in Experiment 1 (Bilalić et al., 2011b). Pictures of full-board chess positions or faces were presented upright or inverted. Participants had to indicate whether the currently presented stimulus matched the previously presented stimulus. **(C)** Stimuli of Experiment 2 (Bilalić et al., 2011a). The stimuli were presented on a 3 × 3 miniature chess board. In the check task participants indicated whether the black piece (knight or rook) gives the white king check. In the identity task participants indicated whether the presented black piece is a rook or a knight. In the control task participants identified geometrical shapes (square or diamond). **(D)** Stimuli of Experiment 3 (Bilalić et al., 2011b). Pictures of full-board chess positions were presented. Participants had to indicate whether the white king was in check in the check task, whether there were knights of both colors presented in the knight task, or whether two dots (black and white) were present in the control (dot) task. **(E)** Stimuli of Experiment 4 (Bilalić et al., 2010, 2012). Pictures of full-board chess positions were presented. Participants had to indicate whether the number of black threats (how many times black can take white) was four in the threat condition, whether the number of knights and bishops was four in the knights and bishops task and whether the number of all pieces on the board was 15 in the control task. In all three tasks of Experiments 3 and 4, there were two types of positions: normal (taken from chess games of masters) and random (pieces were randomly distributed on the board).

In the study of Huberle and Karnath (2012) a global circle or square was constructed from smaller local images of circles or squares. Figure 1A illustrates examples from this set of stimuli. Objects at the global level were scrambled by exchanging a certain percentage of the local images with each other, thereby producing a set of stimuli at scrambling levels of 20-, 40-, 60- and 80%. The behavioral results of a two-alternative forced choice (2AFC) task to report the category of the object at the global level (“global circle” vs. “global square”) showed that in 20% scrambled stimuli the global gestalt was easily perceived (97% Correct) whereas 80%-scrambling almost completely prevented global perception (52% Correct).

The four studies of Bilalić et al. (2010, 2011a,b, 2012) comprised five analyses of complex chess related visual stimuli. We combined the data from two analyses, originally reported by Bilalić et al. (2010, 2012), because the data of these analyses came from the same imaging sessions with the same subjects. In the following we address this set of data as Experiment 4.

#### Experiment 1 (Bilalić et al., 2011b)

Participants indicated if the current stimulus was the same as the previous one. There were four classes of stimuli: chess and face stimuli, which were presented upright or upside-down (Figure 1B). The face stimuli were black-white pictures of students (Leube et al., 2001, 2003). The chess stimuli were full-board positions taken from a four-million-chess-game database (ChessBase Mega Base 2007, ChessBase GmbH, Hamburg, Germany; www.chessbase.com). Stimuli from four categories (faces upright, chess upright, faces inverted, chess inverted) were presented in blocks of five stimuli. A single stimulus lasted for 2.75 s and was followed by a random noise mask for 0.25 s. A baseline (gray screen with a central fixation cross) was presented at the beginning, after each block, and at the end of the experiment for 14 s. All four conditions were presented four times in each of three runs (12 blocks of each condition in all runs).

#### Experiment 2 (Bilalić et al., 2011a)

This experiment featured three tasks. In the check task, participants indicated if the white king was attacked (i.e., given check) by the only present black piece. There were four different stimuli with two pieces on a 3 × 3 miniature chess board (Figure 1C). The white king was always on the first square of the upper left corner, while the identity of the other piece (knight or rook) and its location (middle of the lower row or the end of the upper row) varied. In the Identity task, participants were presented with the same stimuli as in the check task, but this time they identified the black piece presented. In the non-chess control task, chess pieces had been exchanged by gray-colored geometrical shapes (a circle for the king; a diamond and square for knight and rook, respectively). In parallel to the two chess tasks, the identity (diamond or square) and position (middle of the lower row or the end of the upper row) of the target stimulus were varied, and participants indicated its shape. Stimuli were presented in a block design. There were four runs and 12 blocks in each of them (four blocks for each condition in a single run). The runs were block-randomized and counterbalanced across participants. The experiment started with an empty 3 × 3 board (baseline) for 13.5 s and was followed by a written instruction for 3 s indicating the task type (check, identity, or control). After the instruction an empty 3 × 3 board was presented for 1.5 s. After 1 s a black center cross appeared and was presented for 0.5 s to warn participants about the upcoming stimulus. The following stimulus lasted for 2 s. There were four trials (stimuli) in a block, and after each block the baseline was presented.

#### Experiment 3 (Bilalić et al., 2011b)

These tasks were similar to the previous experiment—recognizing if the white king was in check (check task), recognizing if knights of either color were present (knight task), and recognizing if a dot of either color was presented (dot task—see Figure 1D). The stimuli, however, consisted of full chess positions (containing 15–18 pieces) presented on a full 8 × 8 square chess board. There were two types of positions—normal and random. The normal positions were taken from the same ChessBase database as in Experiment 1 and were typical middle-game positions of master games not previously known to the participants. The random positions were generated by distributing the pieces on the board randomly using the rule that any piece of either color can occur on any square (Vicente and Wang, 1998; Gobet and Waters, 2003). There were four runs with 12 blocks each, comprising two blocks per condition (3 tasks × 2 position types) in a single run. The runs were block-randomized and counterbalanced across participants. The experiment started with a gray screen with a black center cross, which lasted 5–10 s, immediately followed by the instruction for 2.5 s, after which the actual block started. The stimulus was presented for 4 s and was followed by a mask made of a scrambled chess position, which lasted for 0.5 s. There were four trials (stimuli) in each block, and baseline was always presented after each block.

#### Experiment 4: (Bilalić et al., 2010, 2012)

In this experiment full chess boards with 15–18 pieces were presented in normal and random positions. New middle-game positions were sampled from the ChessBase database. The tasks involved enumerations of chess pieces and their relations (Figure 1E). In the threats task, players indicated whether the number of threats (black to white) was four. In the knights and bishops condition, the task was to indicate whether the number of knights and bishops of both colors was four. Finally, in the non-chess control task, all pieces regardless of color or type were counted (indicate if the number is 15).

There were six runs, two for each task. There was only one task (e.g., threats task) in a single run. In one run, 10 meaningful and 10 meaningless stimuli were presented randomly. The runs were block-randomized and counterbalanced across participants. We first presented a starting board (all pieces at their initial location) with a fixation cross as a baseline with jittered duration (6–10 s). After a short gap (0.5 s), the target stimulus was presented until response, followed by the baseline of the next trial. Before the actual sessions, participants were given two practice trials for each task. The reaction time (i.e., the time to complete the task) was the time between stimulus onset until the participant pressed the button.

In all experiments, the stimuli were projected on a screen above the head of the participant via a video projector placed in the adjacent room. The setup resulted in a visual field of 14.6° for the whole scene. Participants saw the stimuli through a mirror mounted on the head coil and indicated their decision by pressing one of two buttons of an MRI-compatible response device held in their right hand.

### MRI acquisition

All fMRI data were acquired using a 3-T scanner (Siemens Trio) with a 12-channel head coil at the University Hospital of Tübingen. All measurements covered the whole brain using standard echo-planar-imaging (EPI) sequences. For the experiments of Bilalić et al. (2010, 2011a,b, 2012) the following parameters were used: TR = 2.5 s; FOV = 192 × 192 mm; TE = 35 ms; flip angle: 90°; matrix size = 64 × 64; 36 slices with thickness of 3.2 + 0.8 mm gap resulting in voxels with a resolution of 3 × 3 × 4 mm^3^. The study of Huberle and Karnath (2012) used the following parameters: TR = 2 s; FOV = 192 × 192 mm; TE = 40 ms; flip angle: 90°; matrix size = 64 × 64; 24 axial slices with a thickness of 5 mm.

### Functional MRI data analysis

The imaging data of Huberle and Karnath (2012) were originally processed using Brain Voyager®, whereas the data of Bilalić et al. (2010, 2011a,b, 2012) were analyzed using SPM5 (Wellcome Department of Imaging Neuroscience, London, UK; http://www.fil.ion.ucl.ac.uk/spm). Brain Voyager® and SPM differ from each other in some crucial aspects. For example, volume normalization in BrainVoyager® transforms the data to the Talairach space whereas normalization in SPM is based on templates in MNI space (Goebel et al., 2006; Lancaster et al., 2007). For the sake of a direct transfer between the studies we re-analyzed the dataset of Huberle and Karnath (2012) using SPM8 (Wellcome Department of Imaging Neuroscience, London, UK; http://www.fil.ion.ucl.ac.uk/spm). Only for the data of Huberle and Karnath (2012) temporal offsets of slice acquisition were accounted for by a temporal realignment. For both studies further preprocessing included spatial realignment of all images of a subject to the mean functional image for motion correction. Only in the studies by Bilalić et al. (2010, 2011a,b, 2012) residual motion artifacts induced by a susceptibility-by-movement interaction were additionally addressed using the unwarp function of SPM5. The mean EPI and all functional images were co-registered to the anatomical image for every participant. All images were normalized using the respective T1-weighted template and smoothed with a FWHM of 8 mm. Modeling of the time series of hemodynamic activation was based on the canonical response function as implemented in SPM5 and SPM8. A high-pass filter with a cut-off of 128 Hz eliminated low-frequency noise components and a correction for temporal autocorrelation in the data was applied using an autoregressive AR(1) process.

**Figure 2:**
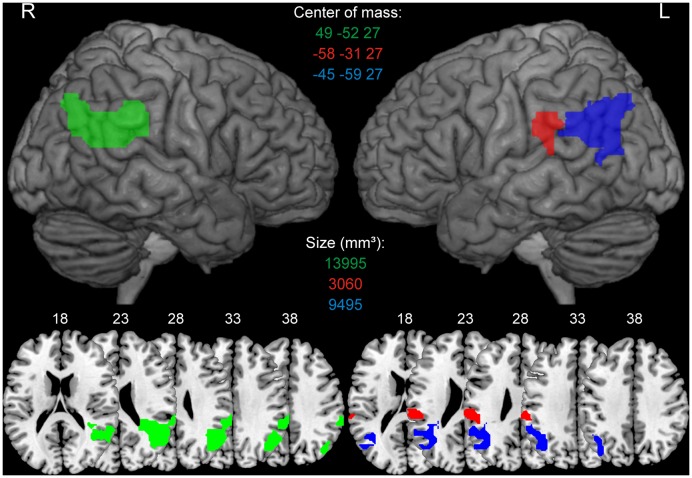
**Regions of interests (ROIs) derived from Huberle and Karnath (2012).** ROIs were identified as those voxels that showed significantly higher BOLD signals for 20%-scrambled objects (intact global perception) compared to 80%-scrambled objects (disturbed global perception) based on a voxel-level threshold of *p* < 0.001 (uncorr.). The green color indicates the right TPJ ROI, red describes the left anterior TPJ ROI, blue indicates the left posterior TPJ ROI. ROIs are presented on a 3D rendered surface and axial slices for the left and right hemisphere. MNI coordinates of the center of mass and size of every ROI in mm^3^ is denoted in the corresponding color.

In the re-analysis of the Huberle and Karnath (2012) data, predictors for each experimental condition were constructed by a convolution of stimulus onsets for 20-, 40-, 60- and 80%-scrambled objects with the hemodynamic response function. The resulting design matrices comprised 4 experimental regressors, one for each scrambling level. Additionally, we included six covariates to capture residual movement-related artefacts. We used the individual participants' contrast images obtained from the first-level analysis for the second-level analysis. Areas involved in the intact perception of global gestalt were identified as those voxels that showed significantly higher signals for 20%-scrambled objects (intact global perception) compared to 80%-scrambled objects (disturbed global perception) based on a voxel-level threshold of *p* < 0.001 (uncorr.) with a cluster extent of at least 50 voxels in the area of the TPJ. We used a relatively liberal threshold to get bilateral ROIs and extend the analysis also to a left-sided TPJ area that was delineated using the same methods and thresholds that served for the right hemisphere. The results of this re-analysis were topographically consistent with the original results produced with Brain Voyager®. However, because of small differences in the statistical procedures between both analysis packages the extent of individual clusters based on individual thresholds were slightly different. The individual clusters that resulted from the re-analysis and which were used for the later ROI analyses are specified in Figure 2. In the further analyses we will label the three ROIs according to their localization as right, left anterior and left posterior TPJ ROI.

In the first two experiments of Bilalić and colleagues (Bilalić et al., 2011a,b), all trials were modeled with their full duration. In Experiment 3 (Bilalić et al., 2011b) the first second and in Experiment 4 (Bilalić et al., 2010, 2012) the first three seconds of each trial were used in order to keep the duration for each condition constant. The rest of the trial was also explicitly specified as a nuisance regressor, while the baseline was implicitly modeled. The mean percent signal changes (PSC) within each ROI were extracted for each participant and condition using MarsBar (http://marsbar.sourceforge.net). The PSC was calculated by dividing the maximum of the time course of the respective estimated event for this condition by the beta value for the constant session mean regressor. PSC values from experts and novices for the respective experiments and conditions were then analyzed with repeated measures ANOVAs.

## Results

In all experiments, chess experts showed a clear behavioral advantage compared to novices for chess related stimuli but not for the control stimuli (for details see: Bilalić et al., 2010, 2011a,b, 2012). To have an overview over all experiments and the respective results see Table 2.

**Table 2:** **Results of statistical comparisons between experts and novices are indicated by a ‘+’ if a significant difference for the respective experiment and factor combination was observed and ‘0’ if the difference was not significant**.

**Experiment task**	**1 N-Back**		**2 Detection (mini chess board)**			**3 Detection (full chess board)**			**4 Counting**		
**Conditions**	**Chess**	**Faces**	**Check/no check**	**Rook/knight**	**Control**	**Check/no check**	**Identity**	**Control**	**Threats**	**Knights & bishop**	**Control**
**TPJ RIGHT**											
Result	+	0	0	0	0	+	+	+	+	+	0
**TPJ LEFT ANTERIOR**											
Result	+	0	0	0	0	+^*^	0	0	+	+	0
**TPJ LEFT POSTERIOR**											
Result	+	0	0	0	0	+^*^	0	0	0	0	0

The asterisks mark significant results derived from preceding full factorial analyses with p-values slightly above 0.05 (please see results section for Experiment 3).

### Experiment 1

For Experiment 1 we calculated a 2 × 2 × 2 repeated measures ANOVA with the following factors and levels: expertise (expert vs. novice) × task (chess vs. face) × presentation (normal vs. inverted presentation).

#### Right TPJ

**Figure 3:**
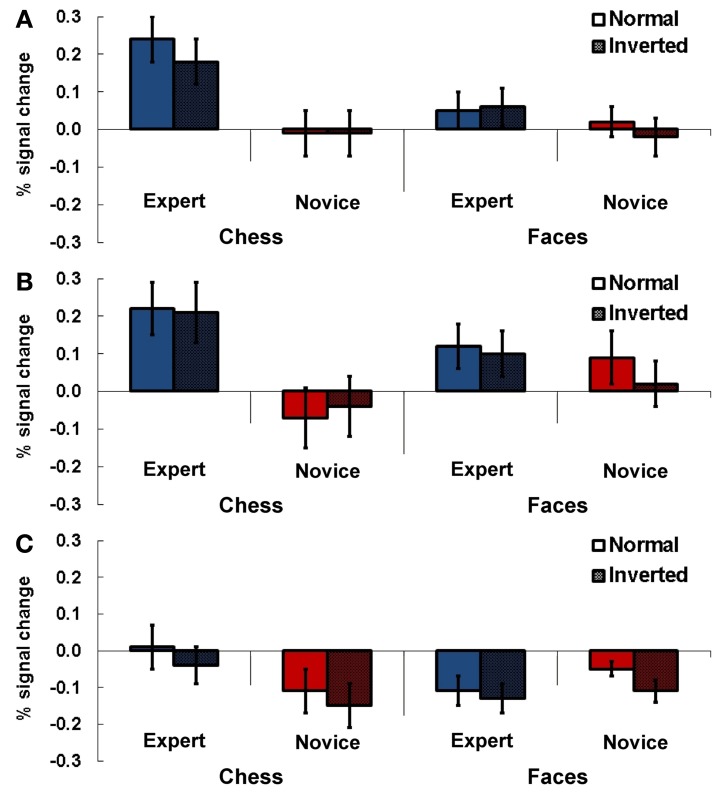
**Results Experiment 1.** Percent signal change (PSC) for the two experimental conditions chess and faces (normal and inverted presentation) for experts and novices. Subjects had to indicate whether the currently presented stimulus matched the previously presented stimulus. Results are presented for TPJ ROI right **(A)**, left anterior **(B)** and left posterior TPJ **(C)**. Error bars indicate standard error of the mean.

Experts showed stronger activation in the right TPJ area compared to novices depending on the stimulus category administered in the particular tasks (Figure 3A). Significantly stronger activations were evident for chess related stimuli in experts, while we found no significant difference between experts and novices for faces. The statistical analysis showed a significant main effect for task [*F*_(1, 13)_ = 6.74, *p* = 0.02, η^2^_*p*_ = 0.34] and a significant interaction effect for the factors expertise and task [*F*_(1, 13)_ = 8.92, *p* = 0.01, η^2^_*p*_ = 0.41]. Two separate Two-Way ANOVAs for the two tasks (chess/faces, with factors presentation and expertise) showed a significant main effect for expertise for chess related stimuli [*F*_(1, 13)_ = 7.14, *p* = 0.02, η^2^_*p*_ = 0.36] while no effect was observed in the control condition [faces, main effect expertise: *F*_(1, 13)_ = 0.86, *p* = 0.37, η^2^_*p*_ = 0.06]. In these analyses, there was no effect involving the factor presentation (*p* > 0.12).

#### Left anterior TPJ

In the anterior left TPJ ROI a similar result pattern emerged. There was a stronger activation in this region for experts than in novices depending on the stimulus material administered (Figure 3B). In this ROI experts also showed stronger activations for complex chess related stimuli, while no meaningful difference between experts and novices was observable for faces. This was approved by the statistical analysis: a Three-Way ANOVA showed a significant interaction effect for expertise and task [*F*_(1, 13)_ = 15.09, *p* = 0.002, η^2^_*p*_ = 0.54]. The following separate ANOVAs for the two tasks revealed a significant main effect for expertise for chess stimuli [*F*_(1, 13)_ = 7.50, *p* = 0.017, η^2^_*p*_ = 0.37], while a significant main effect in the faces condition was present for the factor presentation only [F_(1, 13)_ = 6.67, *p* = 0.02, η^2^_*p*_ = 0.34].

#### Left posterior TPJ

For the posterior left TPJ ROI the previous result pattern was not observable (see Figure 3C). The Three-Way ANOVA showed a significant main effect for presentation [*F*_(1, 13)_ = 16.99, *p* = 0.001, η^2^_*p*_ = 0.57] and a significant interaction effect for the factors task and expertise [*F*_(1, 13)_ = 8.68, *p* = 0.011, η^2^_*p*_ = 0.40]. In the subsequent Two-Way ANOVAs for the two different tasks (chess/faces) a significant main effect for presentation was observable in the chess task [*F*_(1, 13)_ = 5.28, *p* = 0.039, η^2^_*p*_ = 0.29] while no effect was present for faces [*F*_(1, 13)_ = 2.84, *p* = 0.12, η_*p*_^2^ = 0.18].

### Experiment 2

**Figure 4:**
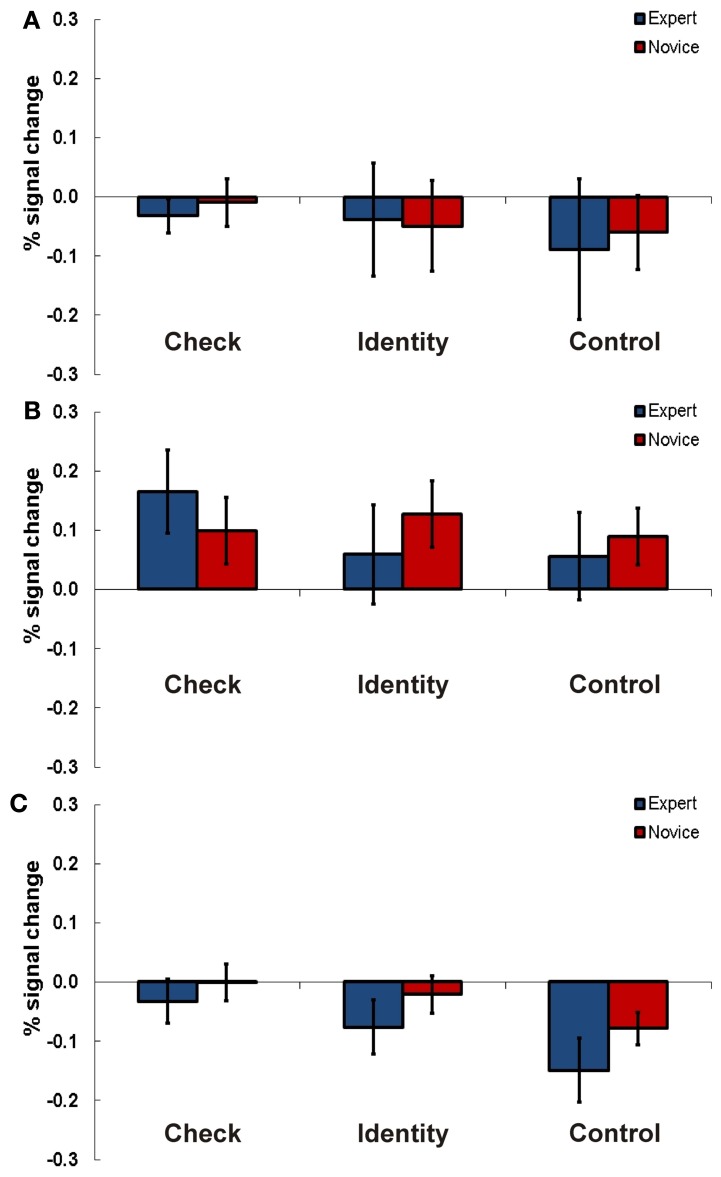
**Results Experiment 2.** Percent signal change (PSC) for the three experimental conditions check (indicate if knight is in check), identity (recognition of a chess piece), and control (recognition of a geometrical shape) for experts and novices in TPJ ROI right **(A)**, left anterior **(B)**, and left posterior TPJ **(C)**. Error bars indicate standard error of the mean.

For Experiment 2 a 2 × 3 repeated measures ANOVAs with the factors expertise (expert vs. novice) and task (check vs. identity vs. control) were calculated for each ROI. These analyses did not reveal any differences between experts and novices (see Figure 4).

#### Right TPJ

For the right TPJ region we found a significant main effect for task [*F*_(2, 28)_ = 4.44, *p* = 0.021, η^2^_*p*_ = 0.24].

#### Left anterior TPJ

Also in the anterior left TPJ area we found a significant main effect for task [*F*_(2, 28)_ = 3.63, *p* = 0.04, η^2^_*p*_ = 0.21]. Additionally, the interaction of task and expertise was significant [*F*_(2, 28)_ = 4.68, *p* = 0.015, η^2^_*p*_ = 0.26]. *Post-hoc t*-tests looking for significant differences between experts and novices in the three tasks did not show any significant results.

#### Left posterior TPJ

In the posterior left TPJ region the Two-Way ANOVA showed a significant main effect for task as well [*F*_(2, 28)_ = 15.98, *p* = 0.001, η^2^_*p*_ = 0.53].

### Experiment 3

In this particular experiment a 2 × 3 × 2 design was used. It contained the following factors and levels: expertise (expert vs. novice) x task (check vs. knight vs. dot) x position (normal vs. random).

#### Right TPJ

**Figure 5:**
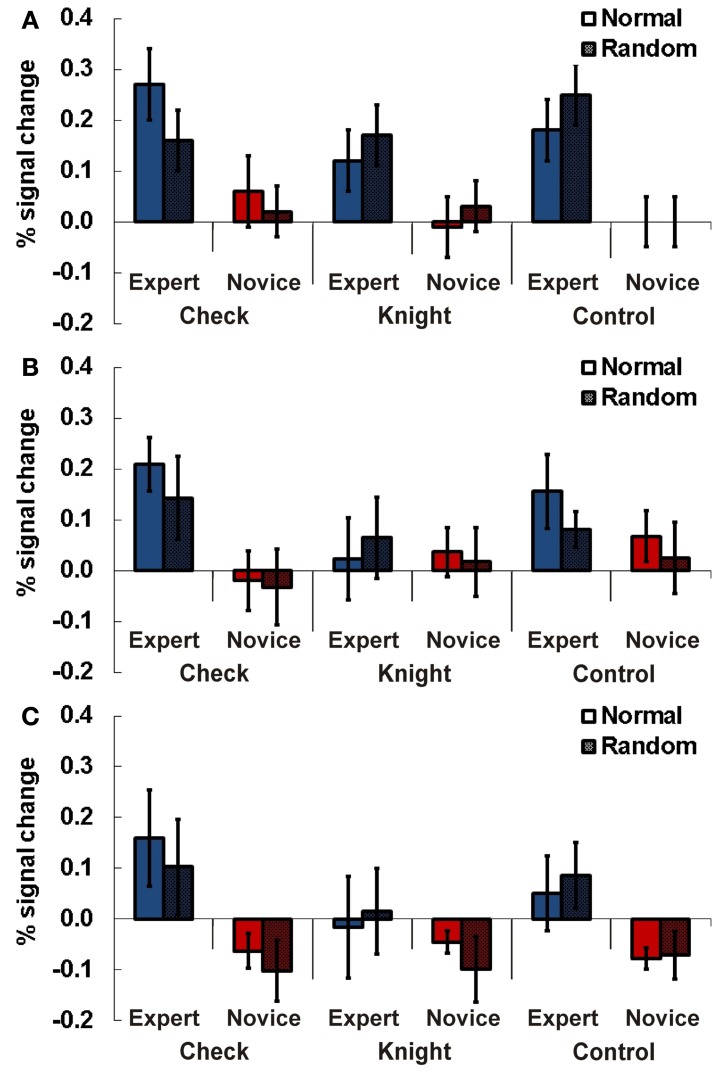
**Results Experiment 3.** Percent signal change (PSC) for the three experimental conditions check (recognizing if the white king is in check), knight (recognizing if black/white knights are present), and control (recognizing if a black/white dot is presented) for experts and novices in normal (chess pieces arranged according to real chess matches) and random (chess pieces in randomized distribution) chess arrays. Results are presented for TPJ ROI right **(A)**, left anterior **(B)**, and left posterior TPJ **(C)**. Error bars indicate standard error of the mean.

In the right-hemispheric TPJ region experts showed stronger activations compared to novices across all three tasks (see Figure 5A). A Three-Way ANOVA including all factors confirmed this observation by a significant main effect for expertise [*F*_(1, 13)_ = 7.70, *p* = 0.016, η^2^_*p*_ = 0.24]. We observed a slightly non-significant interaction effect for the factors task and position [*F*_(1, 13)_ = 3.82, *p* = 0.07, η^2^_*p*_ = 0.19]. No other main effects or interactions were significant (all *p* > 0.28).

#### Left anterior TPJ

In the anterior left-hemispheric ROI the Three-Way ANOVA revealed an interaction effect for the factors expertise and task just above the adopted type-1 error probability threshold of 0.05 [*F*_(2, 26)_ = 3.12, *p* = 0.06, η^2^_*p*_ = 0.20, see Figure 5B]. Subsequent separate 2 × 2 ANOVAs for the different tasks showed a significant main effect for expertise in the check task [*F*_(1, 13)_ = 5.12, *p* = 0.042, η^2^_*p*_ = 0.28].

#### Left posterior TPJ

The analysis for the posterior left-hemispheric ROI also revealed an interaction effect for the factors expertise and task slightly above the probability threshold [*F*_(2, 26)_ = 3.23, *p* = 0.056, η^2^_*p*_ = 0.20, see Figure 5C]. Separate 2 × 2 ANOVAs for the different tasks demonstrated a significant main effect for expertise in the check task [*F*_(1, 13)_ = 4.78, *p* = 0.048, η^2^_*p*_ = 0.27].

### Experiment 4

For Experiment 4 a 2 × 3 × 2 design was applied. It comprised the following factors and levels: expertise (expert vs. novice) × task (threat vs. knight & bishop vs. control) × position (normal vs. random).

#### Right TPJ

**Figure 6:**
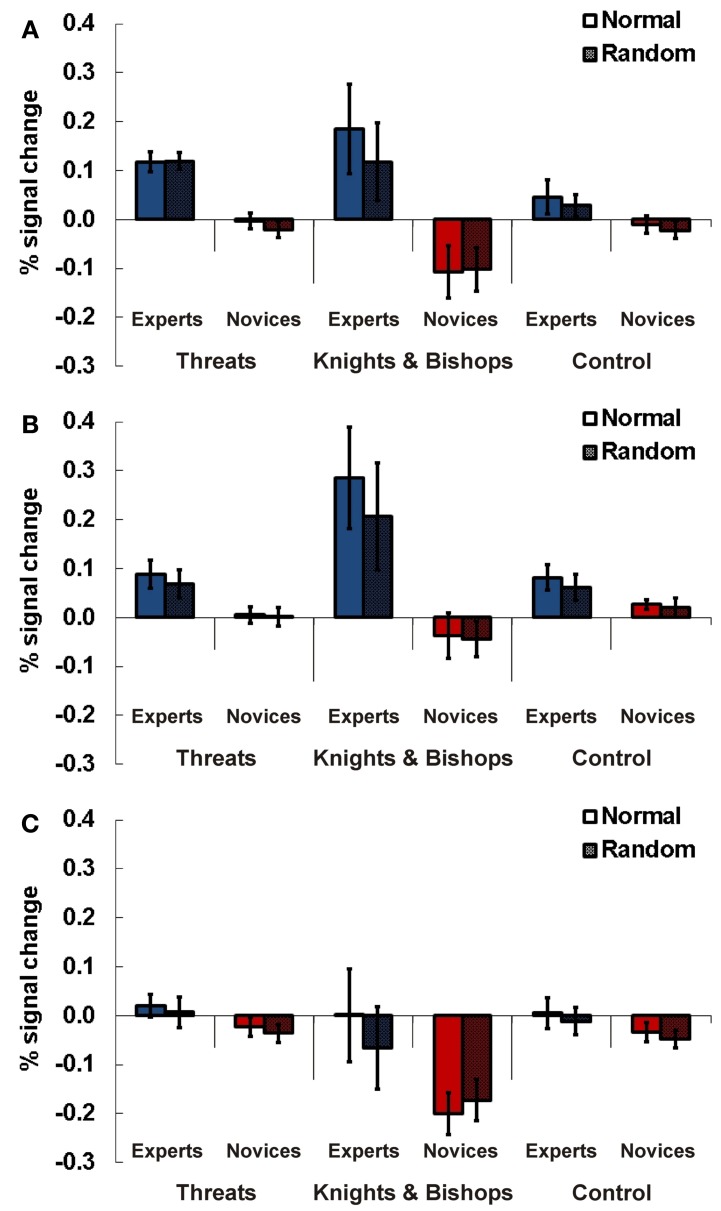
**Results Experiment 4.** Percent signal change (PSC) for the three experimental conditions threats (indicated whether the number of threats of black to white was four), knights and bishops (indicate whether the number of knights and bishops of both colors was four), and control (non-chess control task, all pieces regardless of color or type were counted, indicate if the number is 15) for experts and novices in normal (chess pieces arranged according to real chess matches) and random (chess pieces in randomized distribution) chess arrays. Results are presented for TPJ ROI right **(A)**, left anterior **(B)**, and left posterior TPJ **(C)**. Error bars indicate standard error of the mean.

In the right-hemispheric TPJ region experts compared to novices showed higher signals for chess related stimuli than for control material (see Figure 6A). This result was confirmed by a Three-Way ANOVA showing a significant main effect for expertise [*F*_(1, 21)_ = 13.19, *p* = 0.002, η^2^_*p*_ = 0.37] and an interaction effect for the factors expertise and task [*F*_(2, 42)_ = 5.18, *p* = 0.01, η^2^_*p*_ = 0.20]. In separate ANOVAs for every task (threat, knight & bishop, control) significantly higher activations for complex chess stimuli were confirmed for chess experts compared to novices. The main effect for expertise was significant for the threat [*F*_(1, 21)_ = 29.24, *p* < 0.001, η^2^_*p*_ = 0.58] and the knight & bishop task [*F*_(1, 21)_ = 8.68, *p* = 0.008, η^2^_*p*_ = 0.29], but slightly not for the control task [*F*_(1, 21)_ = 3.65, *p* = 0.07, η^2^_*p*_ = 0.15].

#### Left anterior TPJ

For the anterior left-hemispheric TPJ region we observed a similar result pattern. Experts compared to novices showed stronger neuronal activations for chess related stimuli than for control material (see Figure 6B). A Three-Way ANOVA confirmed this observation with a significant main effect for expertise [*F*_(1, 21)_ = 12.42, *p* = 0.002, η^2^_*p*_ = 0.40] and a significant interaction effect of expertise and task [*F*_(2, 42)_ = 5.89, *p* = 0.006, η^2^_*p*_ = 0.24]. The subsequent Two-Way ANOVAs for the three tasks revealed significant main effects for the factor expertise for the threat [*F*_(1, 21)_ = 5.54, *p* = 0.029, η^2^_*p*_ = 0.23] and the knight & bishop task [*F*_(1, 21)_ = 10.20, *p* = 0.005], while no effect was present for control material [*F*_(1, 21)_ = 1.85, *p* = 0.19, η^2^_*p*_ = 0.35].

#### Left posterior TPJ

In the posterior left TPJ we found a significant three-way interaction for expertise x task x position [*F*_(2, 42)_ = 4.12, *p* = 0.024, η^2^_*p*_ = 0.18, see Figure 6C]. Subsequent ANOVAs for every task showed a significant interaction for position and expertise [*F*_(1, 21)_ = 4.39, *p* = 0.05, η^2^_*p*_ = 0.19] in the knights & bishop task. The following *post-hoc t*-tests supported a difference between experts and novices for normal [*T*_(21)_ = 2.32, *p* = 0.03, Cohen's *d* = 0.95] but not random presentations [*T*_(21)_ = 1.29, *p* = 0.21, *d* = 0.59]. However, the difference for normal presentations barely missed the priorily adopted significance threshold of *p* = 0.05 after Bonferroni correction.

## Discussion

In a series of four independent ROI analyses the BOLD signal changes in bilateral TPJ areas during the perception of complex chess related visual stimuli were investigated. We examined possible neuronal differences between chess experts and novices in left and in right TPJ regions that were associated with localized signal increases in an independent experiment on global perception (Huberle and Karnath, 2012). We hypothesized that superior visual processing skills for highly familiar, complex material (expert view) are strongly associated with enhanced visual integration abilities: chess experts perceive chess situations rather at the global level (full chess board) whereas novices focus on the local level (individual chess pieces). Indeed, experts compared to novices showed higher signals in bilateral TPJ areas during the presentation of complex but highly familiar chess stimuli in three of four ROI analyses. These signal differences were consistent for all stimuli with meaningful, chess-related content. Furthermore, the absence of significant differences between experts and novices in experiment 2 is in good agreement with our hypothesis. The stimuli used in Experiment 2 (Bilalić et al., 2011a; see Figure 1C) displayed a simplified version of a checkerboard with three by three fields. In the other experiments stimuli consisted of full chess boards and multiple chess pieces in various configurations (Bilalić et al., 2010, 2011b, 2012; see Figures 1B,D,E).

Our observations strengthen previous data that suggested a significant role of the TPJ in the processing of complex object configurations (Huberle and Karnath, 2012). This assumption is in good agreement with our current knowledge about the typical bilateral area of damage or degeneration in patients with simultanagnosia (Rizzo and Hurtig, 1987; Friedman-Hill et al., 1995; Rafal, 1997; Karnath et al., 2000; Tang-Wai et al., 2004; Valenza et al., 2004; Huberle and Karnath, 2006; Huberle et al., 2010; Thomas et al., 2012). Against the assumption that TPJ plays a specific role for global perception it might be argued that it simply controls attentional switches between or the balancing of local and global visual inputs. Mosaic stimuli like the ones used by Huberle and Karnath (2012) would allow for a detection of global shapes by low scale visual feature detectors early in the visual system, balanced with information coming from high scale visual feature detectors by the TPJ. However, the observation of similar signal changes in a set of experiments using chess board stimuli argues against this interpretation. The relationships between the local items, i.e., chess pieces, are not created through physical features but through semantic relations between the local stimuli whereas the physical characteristics are substantially different from the typical stimuli used in studies on visual integration (e.g., Fink et al., 1996; Huberle and Karnath, 2012). Thus, we assume that our observation of consistent signal changes at the TPJ in two experimentally very different situations with substantially different stimulus material supports a role of TPJ in visual integration processes beyond attentional control.

The ROIs for the right and left hemisphere analyzed in the present study were different. Whereas a single ROI was analyzed for the right hemisphere, two separated ROIs were used for the left hemisphere. This was the consequence of the transfer of the functional definition of these ROIs from the preceding global perception experiment (Huberle and Karnath, 2012) to the chess expert datasets based on accepted voxel- and cluster-level thresholds. Thus, the definition of these regions was based on objective statistical criteria to allow reproducibility. Obviously, using other voxel- and cluster-level thresholds or slightly different first- and second-level statistics might have resulted in somewhat different delineations of the ROIs. However, the general pattern of the results would not differ. The signal patterns in the analyzed ROIs suggested a relative lateralization of visual integration to the right hemisphere. We found strong interaction effects including the factor expertise for the large right TPJ ROI, whereas only the anterior left TPJ ROI revealed consistent differences between experts and novices during the presentation of complex visual material across Experiments 1, 3, and 4. In contrast, the more posterior left TPJ region showed much less consistent results with a somewhat conclusive signal pattern only for Experiment 3. The idea of a relative lateralization that was not tested explicitly would be in agreement with several studies arguing for a right hemispheric specialization for global aspects in visual integration (Martin, 1979; Robertson et al., 1988; Fink et al., 1997b; Yamaguchi et al., 2000) and perception of complex chess configurations (Krawczyk et al., 2011). Nevertheless, one study reported a left hemispheric dominance for processing of global features of complex visual material (Fink et al., 1997b). This variability between fMRI studies depending on the particular task and samples may also indicate that global perception processes are bilaterally represented in the human left and right hemispheres. The fact that the vast majority of the patients showing simultanagnosia suffered bilateral brain damage (Rizzo and Hurtig, 1987; Friedman-Hill et al., 1995; Rafal, 1997; Karnath et al., 2000; Tang-Wai et al., 2004; Valenza et al., 2004; Huberle and Karnath, 2006, 2010; Thomas et al., 2012) supports this assumption.

The observed association of superior skills with an increased BOLD signal in a confined cortical structure is also in line with studies investigating the neuronal effects of visual perceptual training and expert view in other brain regions. However, in numerous functional imaging studies on perceptual learning it was demonstrated that training results in higher BOLD signals in task-related brain areas (Gauthier et al., 1999; Grill-Spector et al., 2000; Furmanski et al., 2004; Op de Beeck et al., 2006; Jastorff et al., 2009). Particularly in the context of global perception, an increase of BOLD signal amplitudes was associated with an improvement of complex stimulus processing through learning (Maertens and Pollmann, 2005; Zhang and Kourtzi, 2010; Zhang et al., 2010; Mayhew et al., 2012). Beyond, research on expert-novice differences showed higher BOLD signals in experts (Gauthier et al., 2000; Rhodes et al., 2004; Harley et al., 2009). Our observations may also be addressed to prolonged training effects causing modulation and fine-tuning of other non-visual areas (Moore et al., 2006; Guida et al., 2012). We therefore suggest that the increase of the BOLD signal in the TPJ represents an important contribution to the behavioral difference between experts and novices. Further, we did not observe any significant effects for inverted presentations or random chess positions, arguing for highly automatized global processing mechanisms for chess configurations in over-trained experts. Whereas other complex visual stimuli like faces become more or less incomprehensible by an inversion, chess boards are still interpretable. Therefore, we did not expect clear-cut inversion effects for global chess stimuli in the TPJ ROIs, similar to the well-established differences for faces in the respective brain areas (Epstein et al., 2006).

However, neuroimaging studies of learning and expertise in other cognitive domains, like visual working memory (WM), showed different or even opposite BOLD result patterns with behavioral changes (Landau et al., 2004; Kelly and Garavan, 2005; Jaeggi et al., 2007). Jaeggi et al. (2007) demonstrated higher BOLD signals in low-performers than in experts in a working memory task. Landau and colleagues (Landau et al., 2004) found that learning led to a decrease of BOLD signals in several cortical brain areas. Obviously, there may exist several other factors, like advantages in working memory or motivation driving neuronal signals in expert view. However, the present study highlights an important contribution of visual integration and the associated neuronal structures to superior visual skills in chess experts.

In conclusion, our data show that fMRI signals in the TPJ are increased during the observation of complex stimuli in experts who experienced an extensive training that most likely resulted in superior skills of visual integration. The results of our cross-paradigm ROI analyses shows that such signal increases are not only observed using highly selective global/local stimulus material in within-subject comparisons but can be detected in between-subject comparisons using stimulus material from a different field of research. In good agreement with previous fMRI studies (Himmelbach et al., 2009; Huberle and Karnath, 2012) and patient reports (Rizzo and Hurtig, 1987; Friedman-Hill et al., 1995; Rafal, 1997; Karnath et al., 2000; Tang-Wai et al., 2004; Valenza et al., 2004; Huberle and Karnath, 2006; Huberle et al., 2010; Thomas et al., 2012) the presented data supports the assumption of a crucial involvement of the left and the right TPJ in global gestalt perception.

### Conflict of interest statement

The authors declare that the research was conducted in the absence of any commercial or financial relationships that could be construed as a potential conflict of interest.
